# Facing the Involvement of Youths in Competitions: Soviet Visions and Adaptations to the Rejuvenation of Elite Sports (Second Half of the 20th Century)

**DOI:** 10.3389/fspor.2020.568025

**Published:** 2020-10-21

**Authors:** Sylvain Dufraisse

**Affiliations:** UMR 6025 CENS, Université de Nantes-CNRS, Nantes, France

**Keywords:** soviet union, history, elite sports, soviet sports, youth sports, history of sports science, cold war sports

## Abstract

This article focuses on Soviet sports authorities' adaptations to youth involvement in elite sports during the second half of the 20th century during the Cold War. It demonstrates that the quest for performance and success in world competitions meant that sportsmen needed to start training at younger ages. This trend led to the development of a biopolitical expertise on youth sports, that mixed scientific research, artistic and intellectual stances and public policy making. It contributed to determining age requirements and a specific system to intensify preparation while protecting the sportsmen involved. Since the mid-1970's, this system was not well-received within the Soviet Union as well as by the wider world. These youth systems embodied the poor Soviet management of childhood.

## Introduction

The Soviet Union's sports administration after 1950 proved its capacity to prepare elite sportsmen. Sports in the Cold War, like music, arts, literature and dance, emerged as symbols of national prestige and were fields of intense political battles. Athletic endeavors could symbolize the superiority of a political system as performances and records, “objective” measures of domination, were diffused worldwide by the media. Soviet champions who stood on world championship podiums and obtained gold medals were flagship representatives of the Soviet Union and gave the state a friendly and cheerful image.

Commentators and journalists were the first to write about Cold War sports. They selected epic “battles” in pools and on pitches, popularized some of the most iconic sports personalities (e.g., Nadia Comaneci), and contributed to shaping a triumphalist Cold War narrative (Mellis, 2019). Sports were for a long time largely absent from mainstream Cold War historiography, apart from mega-events, symbolic battles or climatic moments like the boycotts, reinforcing the US-USSR rivalry and producing what military history called combat history (“histoire-bataille”) (Edelman, 2019; Clastres, 2020; Vonnard and Marston, 2020). Recent works on Cold War sports, based on new archival materials, have explored new paths and shown how relevant sport can be to analyze the Global Cold War. They highlight the “interactions and exchanges in which multiple players were involved” (Del Pero, 2016), accentuating the instances where members of the divided blocs cooperated (Andrews et al., 2006; Vonnard and Quin, 2017; Vonnard et al., 2018) or turning their attention to the role of sports in newly decolonized countries (Edelman, 2019). This scholarship underlines how gender and race became crucial in the symbolic opposition between Capitalist and Communist countries (Malz et al., 2007; Barker-Ruchti, 2009; Bohuon, 2012; Edelman, 2019). It also analyzes how the Cold War and the growing importance given to sports transformed national sports systems and sporting programs and impacted the relations between sports leaders and athletes (Hunt, 2006; Gygax, 2012; Rider, 2017; Dufraisse, 2019; Mellis, 2019). Moreover, these new works explore “the ambiguous zones within which international organizations, states, sports leaders and athletes interacted in order to achieve their respective goals during the Cold War” (Mellis, 2019).

Soviet sport was long considered a realm of totalitarian control, a “Big Red machine.” Recent works eclipse this simplistic interpretation of Soviet sport, which overemphasized the role of the State and of ideology. Scholars interested in Soviet sports, using archives, interviews and local newspapers, have highlighted new aspects that provide a more nuanced understanding about: the diversity of practices within the Soviet Union, the latent debates about the goal attributed to sports and to physical education and the place given to sportsmen, the tense and precarious balance between the sports administration and sports societies, and the role of patrons within the sport system (Edelman, 1993, 2009; Katzer et al., 2010; Grant, 2013; Parks, 2016; Maddox, 2018; Zeller, 2018; Dufraisse, 2019). Critically, this scholarship illustrates that the development of sports performance policies in the Soviet Union was not linear. Instead, it was the fruit of negotiations and struggles between sports societies, party organs and the administration of sports and physical culture. It was not only endogenous and adjusted to scientific and technical contacts and foreign influence from the Western countries, the newly socialist countries or the territories annexed by the Soviet Union (Dufraisse, 2017). Soviet performance-enhancing activities were also exported and adapted abroad (Krüger, 2016).

The Cold War context gave a boost to transnational debates and controversies on the making of Eastern champions. In Western countries, the Communist milieu largely supported the Soviet champions' exploits but some Communists sports leaders, for example in France, expressed doubts and criticisms (Martinache, 2019, 2020). Non-Communist journalists and sports leaders questioned the fairness of Eastern practices and blamed the abuses in sport on socialist systems.

Western media polemics also affected the way sportsmen and sportswomen were groomed and focused on successive issues and abuses: the problem of amateurism, the eagerness of Eastern champions for Western goods and way of life, the unfair play in adherence to Western perceptions of fairplay, the use of doping (Hunt, 2011; Edelman, 2017; Dufraisse, 2019).

The Soviet sports administration was not cloistered and adapted its policies. It also ensured its practices adhered to international federation rules, and to criticisms that appeared in the Western media and in the Soviet public sphere. Facing the rejuvenation of elite sports, the Soviet sports administration tried to control more closely the practices of the young sportsmen involved. At the end of the 1970's and during the 1980's, the involvement of young sportsmen and sportswomen in elite sports and competitions, age falsification and intensive training at a young age became a public problem in the Western countries and in international federations, and a way to criticize Soviet abuses so as to win at any cost^1^ (Cervin, 2017). The question of youth involvement, age requirements and special policies applied to young sportsmen in sports during the 1960's and the 1970's aroused a larger interest due to two tendencies: on the one hand, the rejuvenation of elite sports, the necessity to prepare high-level sportsmen from their very childhood and the will to protect them; on the other hand, a broader issue, the bigger visibility of youth in the public sphere and the necessity to deal better with the specificities of this segment of the population, even in the Soviet Union (Bantigny, 2007; Bantigny and Jablonka, 2009; Tsipursky, 2016).

The issue of performance sports for children must also be observed in the Soviet context. The period from the 1950's to the 1980's was characterized by contradictory trends: the project of developing a more collective upbringing launched by Nikita Khrushchev, the desire to select and support children with high levels of ability, who would be the next vanguard of the motherland, the wish to develop harmonious individual personalities and the tendency to consider children as vulnerable and needing special psychological protection (White, 2020).

This article demonstrates that, confronted with the requirements of performance sports and the necessity to get podiums and medals, many Soviet sports specialists and public authorities tried to improve the conditions of youths involved in sports, to take care of them and to ponder the potential dreadful consequences of it on their bodies and minds. This trend led to the development of a biopolitical expertise on youth sports that mixed scientific research, artistic and intellectual stances and public policy making (Bantigny et al., 2011).

## Materials and Methods

This article examines how elite sport life-cycles were produced, justified and implemented in the Soviet Union, taking into account how global public opinion and discussions in international instances may have influenced internal decisions.

Age can be a medium for categorizing, for governing populations and for organizing the stages of the existence (Mauger, 2010; Bozon and Rennes, 2015; Rennes, 2019). In sports, the effects of the life-cycle and age are determining factors. Age is used to divide official categories; age-based requirements could be implemented to enter training programs or professional careers. Socially, life-cycles shaped one's experience of physical exercise (MacRae, 2016). In sports, age and life cycle concentrate the attention of many agents: national and international rulers and leaders, journalists, physicians, trainers, sponsors, scholars. These protagonists may have contradictory views and opinions on the “age-based” discrimination, on the effects on physical activities on young bodies or on sportsmen's and sportswomen's life-cycles. Contrary to the image that was later developed, the manipulation of Eastern sportswomen's birth date, or that international criticism highlighted, Soviet scientists, physicians and artists worked on the issue of child abuse in sports. Moreover, sports authorities implemented special policies to deal with the rejuvenation of elite sports and increased attention on children's rights. This article draws out discussions and debates that led to regulation of youth elite sports and overcame the common “blame” of the Eastern abuses in elite sport.

To do so, this article follows Antoine de Baecque's proposition of a “non-quantitative serial history” (de Baecque, 1993) and gathers texts that can be read with images and administrative documents that pertain to a common issue. The sources used for this analysis – archives, films, scientific publications – were produced by Soviet institutions from the late 1950's to the 1980's, specifically discussed youth elite sports, and contributed to producing a panoramic vision on how youth elite sports were perceived. The sources examine how a biopolitical expertise on the relations between age and performance was developed in the USSR and illustrate that political demand and funding given to applied research helped to build elite sports and elite youth sports programs.

Three kinds of materials are used to draw out scientific perceptions and prescriptions. Articles from the journal *Problemy ûnošeskogo sporta* (1958–1962), *Teoriâ i Praktika Fizičeskoj Kultury* and conference proceedings on children, sports and physical activities gathered work from specialists of physical activities, sports and children and assembled the Soviet scientific community dealing with child sports. To obtain a more administrative vision on the way child sports were regulated and defined, this article draws on documents from the secretariat of the Sports Soviet administrations, which implemented sports policies and administered sports (*Pansoviet Physical Culture and Sports Committee, Central Union of Sports associations and organizations)*. These documents permit an understanding of how scientific works weighed on political decisions. Also consulted are materials from the Department of Sports and Mass defense works from the Komsomol (Communist Youth organizations). The Komsomol was in charge of the organization of political education in elite sports and tried to diffuse Communist morals to young sportsmen and sportswomen. It also developed a Youth Mass sports program. The Komsomol was quite critical of the elite-oriented approach to Soviet sports and heartily wished to promote a mass sports program. Archival materials were drawn from the Russian State national archives (GARF, *Gosudarstvennyj arhiv Rossijskoj Federacii*) and from the Russian State Archive of Socio-Political History (RGASPI, *Rossijskij gosudarstvennyj arhiv social*′*no-političeskoj istorii*).

Combining these three institutional views on child elite sport illustrates the circulation of ideas on sports public policies from the academic world to the political milieu. But scholars and political representatives were not the only ones to talk about child elite sports. Soviet directors made documentary and fictional films on children's involvement into sports. Until the late 1960's, youths were not the central heroes of films dealing with elite sports. Presenting younger sports heroes was evidently an adaptation to the growing production of a Soviet mass culture and to the rejuvenation of the public. Juxtaposing the narratives of eight Soviet films – documentary and fictional –released between 1968 and 1988, this article intends to illustrate the evolution and the plurality of discourses on child and youth performances. Importantly, these films are used as historical material and not as a pure representation of reality. Conscious that a plurality of agents and social interests are involved in making films, these films illustrate how cinema can give a “form to history” and “make it visible” (de Baecque, 2007). The depreciative visions of child elite sports appeared in the Soviet Union before criticism on child elite sports and Eastern abuses appeared on the international sports scene. Thus, these films are used as traces and examples of discordant discourses on child elite sports.

This article is also inspired by works that examine Soviet policies in a transnational configuration (David-Fox, 2011), such as childhood questions (Denéchère and Droux, 2015; Niget and Denéchère, 2015). The defense of children's rights was the subject of transnational mobilizations. That's why French and American newspaper extracts are used to point out how these issues were addressed in world-ranging newspapers, as well as in polemical books and in journals specialized in sports and physical activities. In doing so, the international polemic about Soviet child abuse is cast within the national and international context of their breaking.

This article is divided into four main sections. The first section analyzes how youth sports were organized and administered in the Soviet Union. The second section focuses on the emergence of a biopolitical expertise on child elite sports in the Soviet Union. These two sections highlight how children and teenagers' involvement in sports was presented, then accepted, discussed or contested in the administrative organs and the academic milieu. The third section studies eight Soviet films. If some documentary films present Soviet child involvement in elite sports as a necessary step of the ‘coubertinian pyramid’ (the mass leads to the elite), others could expose a dissonant vision of child elite sports. The fourth section analyzes how Soviet youth involvement in sports, particularly in gymnastics, became a symbol of the derives of the Socialist countries' management of childhood, while international organizations, journalists and specialists of physical activities began to care more about the situation of young sports prodigies.

The transliteration ISO 9:1995 was used to romanize the Russian language from the Cyrillic script into the Latin script.

## Results

### Structuring Child Elite Sports: Soviet Responses

Since 1934, the All-Union Physical Culture and Sport Committee instituted the “Be ready for Labor and Defense” (*Bud' Gotov k trudu I oboron'e*) program to promote sport for schoolchildren. This sports program aimed at forming the basis of a movement for national fitness and at improving the physical abilities of youths. The main goal of the BGTO program was clearly to spread the practice of sports and to popularize it. Child competition was not the main issue (Riordan, 1977). Soviet material proved that the question of elite youth sport was on the sports administration's political agenda since the late 1950's. The transformation that happened in the realm of sports has to be analyzed alongside the broader evolution of the conception of, and the attention to, childhood in the postwar Soviet Union. This section will examine the responses of Soviet institutions (the Sports Committee, the Sports science scholars) and the way they contributed to shaping new regulations.

#### The Rejuvenation of the Sportsmen Involved in Elite Sports

In 1960, the Bulgarian scholar Dimitr Mičev published an article in the Soviet sports science journal *Teoriâ i Praktika Fizičeskoj Kultury* in which he analyzed the age of 8,867 sportsmen and 1,426 sportswomen who took part in the Olympic Games from 1896 to 1960. The data he collected showed that very few young sportsmen competed in the Games until 1960: only 3 13-year-old and 5 14-year-old boys (in swimming, sailing, water polo and rowing) (Mičev, 1960). From 1960, the situation clearly started evolving in the Soviet Olympic team, as in others. Russian sociologist Oleg Milštejn compiled the composition of the Soviet teams and demonstrated that athletes were increasingly younger: in Helsinki in 1952, the average age of Soviet Olympians was 32 years old. At Montreal in 1976, it was 23 years old^2^. Nevertheless, the age of the athletes varied from discipline to discipline. The Komsomol archives held statistics from all the Soviet selections preparing for the 1968 Mexico Games. That year, the average age of the track and field team was 27.1 with only two members under 21. The average age of the boxing team was 25,7, the wrestling team 27.

The rejuvenation was particularly visible in disciplines like women's gymnastics or swimming. At the Mexico Games, 22.7% of the Soviet swimming team was under 16^3^. During this competition, the average age of women's gymnasts was 19 years and 10 months (for the Soviet team, 19 years old^4^). Georgia Cervin had noted that this trend toward youth began counter-intuitively in Western countries and especially in the United States of America. If the average age of the US and the USSR teams was almost 28 in 1952, in 1956, the American age fell under 20 and reached 17.5 in 1976. Many US Olympic gymnasts were aged 15 that year. By comparison, the average age of the USSR women's gymnastics team was 24.5 in 1960 and fell to under 20 only in 1976. Only one gymnast was younger than 15 in Olympic Competitions between 1960 and 1976: Maria Filatova (Cervin, 2015).

The championships were the tip of the iceberg. To perform at the highest level during their twenties, one had to be trained more intensively and for a long time. Consequently, sports careers began earlier. Sports administrators tried to bridge the gap between youth and elite and to institutionalize programs to identify and to develop incoming champions (Papin, 2007; Tallec-Marston, 2012). In the Soviet Union, a wide network of sports schools for youth was developed. Sports schools were authorized in December 1933, first run by sports societies like *Dinamo*. On September 28, 1945, the Council of People's Commissaries (the equivalent of the Council of Ministries) issued a decree which officialized the creation of 80 sports schools for youths (males and females aged 17–23 who could attest to a good level of practice). Two years later, according to the report, they had 13,222 members^5^. But the schools were not sufficient to improve athletes' results and their role was quite blurred: did they exist to extend the participation or to prepare the new sports avant-garde? In 1949, the Council of Ministries approved the creation of another structure: the *high-performance sports school* (Š*kola vyšego sportivnogo masterstva*), where sportsmen were trained more intensively under the control of scholars and physicians^6^. These types of structures were not originally set up to welcome children and teenagers. But in practice, they involved ever-younger athletes^7^.

To face the increased competition in world championships, as well as within domestic competitions, some republican or city sports committees developed during the 1960's sports boarding schools where children and teenagers engaged in high-performance sports while simultaneously receiving an education. The first sports boarding school opened in 1961 in Tashkent^8^. Others followed in Kiev in 1966. The Moscow Sports City Committee took part in the creation of a boarding school^9^. The Ministry of Education, together with the union of sports associations and organizations of the Azerbaijan SSR, opened a national sports boarding school for children from 6 to 16, gathering 690 pupils. The ‘škola-internat’ prepared them for three sports: track and field, gymnastics and football. Teachers from the boarding school were sent across the republic to scout and recruit for skilled children. Gathering young athletes in the same boarding school permitted the harmonization of living conditions, medical control and training processes^10^. These institutional changes were testimonies of the transformation of high-performance sport and of the necessity to adapt to the extension of the sports career. The Ministry of Education and the Committee of Physical Culture and Sport decided to generalize the schools and to organize a countrywide network in 1970^11^. By 1976, 25 of them were recorded within the USSR (Dunstan, 1978). The development of sports boarding schools was also the consequence of education reforms. First, Nikita Khrushchev launched in 1957 the promotion of state boarding schools (Coumel, 2014; White, 2020), Khrushchev wanted to impose a collectivist and meritocratic ideal of state upbringing, aimed at proposing an academic and polytechnical education based on a collective everyday life, and to level living conditions. In the case of the Azeri boarding school, among the 690 pupils, 140 were from rural villages. Secondly, Khrushchev also supported the development of specialized schools for children showing high levels of ability in foreign languages, mathematics, art and music (Dunstan, 1978; Coumel, 2014; White, 2020). In 1978, 29 sports boarding schools gathered 14,246 students^11^.

#### Preserving Children's Virtue Against the Corruptive Power of Money

In the aftermath of the Second World War, the Council of the People's Commissaries, then the Council of Ministries of the Soviet Union, reorganized the way elite sportsmen and sportswomen were remunerated. A unique compensation system was set up between 1945 and 1947. This system aimed to rule a sporting world characterized by a lack of organization by inflating remuneration and a physical culture administration with little influence over powerful sports societies. Stipends, awarded by the Supreme Council of Physical Culture and volunteer sports societies, were distributed from 1947. Directives set the numbers of stipends and leveled revenues among athletes from various disciplines. This was implemented to tackle excessive remuneration. A table of bonuses was established to normalize and to limit the amount of money given to victorious athletes and to control the frequency at which they were given (Dufraisse, 2016). A few years later, reports from the Minister of State control attested to a wide range of irregularities^12^. New categories of problems also emerged. The growing internationalization of Soviet selections and the development and the diffusion of performance sports within the Soviet Union modified the shape of the Soviet sports elite. In 1955, the head of the Sports Committee, Nikolaj Romanov, sent a letter to the Council of Ministers where he explained that many youngsters 17–19 were now involved in performance sports and could receive a financial help to improve their living conditions^13^. In 1956, the deputy director of the defense and sports committee from the Moscow Komsomol sent to the Komsomol Central Committee a report enumerating misdemeanors in sports societies (*Burevestnik, Torpedo*…). He indicated that volunteer sports societies were remunerating child athletes, giving them 600–800 rubles a month. The document cited the case of 10th grader (16–17 years old) V. Polevoj, who was hired as a sport “instructor” in a local Dinamo organization (a hidden way to employ and remunerate sportsmen). His family was well off. But Polevoj didn't do well at school and was very undisciplined, according to the report. He finished 10th grade with difficulties. He misbehaved during a training course, from which he was excluded. After parent pressures and excuses to the collective, Polevoj was able to reintegrate into the training courses. The author concluded:

“In most cases, the stipend appointment is justified by sports results. But the family situation and its wealth are not taken into account. The behavior of the head of the family doesn't permit them [the children] to be brought up properly. It is even the opposite: it often spoils them as it was the case with Polevoj and Solovieva. Recently, Solovieva became snobby and undisciplined as she was feeling like a “star” […] The Defense and the Sports Committee from the Moscow Committee of the Komsomol requests you [the Central Committee of the Komsomol] to clean up the way stipends are appointed to children athletes^14^.”

On July 24, 1957, a decree from the Council of Ministers reorganized the system of 600–800 ruble stipends and authorized child remuneration for promising sportsmen or women. Nevertheless, the awarding of stipends to junior athletes was dependent on the material situation of their families. It also determined specific rights, like annual holidays, access to special clinic hospitals, as well as duties: virtuous behavior during training sessions, investment in academic works and ideological programs, individuals training plans…^15^ On March 25, 1959, the Council of Ministries of the Soviet Union adapted the table of bonuses to the transformations that had occurred in elite sports since 1945. It limited the rewards that could be given to youths in local competitions to between 300 and 500 rubles. A junior USSR record setter would receive a 30-ruble gift^16^. This rule lasted for 20 years, a reform designed to curb the increase of rewards, but one that also had a moral issue. In 1958, just after the FIFA World Cup in Sweden, the Strel'tsov scandal received much media attention. The fall of this popular football player gave journalists the occasion to criticize the poor education of elite athletes and the advantageous conditions youngsters could get from victories. The campaign provided the occasion to denounce honorary titles, rewards and the huge amount of money sportsmen could receive at a young age (Dufraisse, 2019). These reforms illustrated how the Soviet Sport Central Administration had regulated economic issues in sports and had tried to adapt the rewarding systems to practices that were occurring. Rewards were created for some youths to authorize practices that existed but their amounts were limited. Their attributions were controlled by commissions. One year later, a report to the Central Committee of the Komsomol, written after the 1960 Rome Olympic Games, complained about the negative effects of rewarding youths with stipends and attested that the practice of illegal remuneration still existed^17^.

Discussions and proposals to rule youngsters' remuneration attest to their practice in the Soviet Union. But they also prove that the sports administration tried to shield young sportsmen and sportswomen from the ideological vision of the pervasive corruptive power of early remuneration by trying to regulate it. The possibility of youth remuneration might only be justified when it fitted with the Soviet meritocratic and moral ideology. In parallel, scholars also developed knowledge on child sports. Academics began to investigate the process of early specialization, its consequences and the way it could be adapted to fit the characteristics of children.

### Developing a Scientific Knowledge on Child Sports

At the end of the First World War, the first institutes of physical education were established in Moscow and Petrograd. The Moscow Institute (GCOLIFK) and the Lesgaft Institute in Petrograd were given a higher school status. At first, they were mostly oriented toward pedagogical and military instruction and workers' physical education. In 1946, chairs for each sport were created and gathered highly skilled and specialized trainers and scientists. University courses for specialized coaches were opened. Another center contributed to developing research in sports science: the All-Union Scientific Institute of Physical Culture (*Central'nyi naučnyj-issledovatel'skij institut fizičeskoj kul'tury*, after 1966, the *Vsesoûznyj naučnyj-issledovatel'skij institut fizičeskoj kul'tury*, VNIIFK). Knowledge about theories and methodologies of training about science sports (biochemistry, biomechanics, physiology…) increased (Riordan, 1977). Soviet participation in international top-level sport stimulated the development of applied sports sciences. The dispersion of sports specialists from Moscow and Leningrad in republican institutes of physical culture across the USSR helped to educate and to train a new generation of sports specialists. Communities of sports science specialists emerged in the Soviet Union and adopted common scientific practices (organization of all-USSR meetings, publication of autonomous academic journals…) (Ryba and Stambulova, 2018).

Among them, scholars focused their investigations on the influence and consequences of intensive sports practice on athletes from different generations. In their studies, some of them – physicians, pedagogues or biologists – focused on youth sports. The physician R. È. Motilânskaâ published in 1950 and in 1956 *Sport I Vozrast*. She justified her investigations by explaining that younger and older people were involved in sports in the Soviet Union and that physicians could help optimize training and adapt them to age characteristics. Physical training had to be improved to fit with the specificities of each generation. To write her book, she gathered anthropometrical data from 960 boys and 450 girls aged 15–20. She pointed out the consequences of sports practices on the functioning of the body. She also insisted that these observations could help create new guidelines to improve early intensive training (Motylânskaâ, 1956). Alongside S. S. Grošenkov, she led research on the consequences of youngsters' intensive practices. They progressively extended their investigations to younger children and early specialization. They launched a survey to analyze child involvement in competitive sports (gymnastics, track and field, collective sports, skiing, ice-skating and boxing) in four Moscow schools (22, 325, 407 et 545) between 1954 and 1956.

Observing physical culture sections in those schools, scholars wanted to determine the best ways to initiate young Soviets to tactics and sports activities and the conditions to implement early specialization. In the first issue of the academic journal *Problemy ûnošeskogo sporta* published in 1958, they gathered the results of their first inquiries^18^. In this issue, no articles dealt precisely with early specialization. Instead, they discussed a huge spectrum of child sports and physical activities: physical education at school (three articles), training methods and teaching tactics in a variety of disciplines (thirteen articles), *Bud' Gotov k Trudu I oboron'e* diploma (two articles), medical control (two articles), physiology (three articles). One could remark that those inquiries studied younger people and began to analyze how it was possible to teach tactics and techniques to 10- or 11-year-old Soviets.

Two more issues were published in 1961 and 1962 that reflected changed attitudes in a short span of time. The introduction of the 1961 issue proposed huge perspectives. Physical education and sports were a way to improve individuality and bodies, to make them more harmonious and effective, to prepare them to work and to defend the motherland. V. È. Nagornaâ, the issue's editor, used common *fizkultura* mottos. The content differed from the 1958 issue and perspectives set in the introduction [and was instead focused on XX?]. The journal evoked physical education in four articles, training methods in 13 articles (in which two studied early specialization). Five articles were about medical control (one of them about sports boarding school) and seven about physiology. The question of the organization of early training was raised in two disciplines: Nordic Combined and gymnastics. The consequences of teenagers' intensive training were observed in weightlifting.

These articles justified the necessity to develop medical control and to make physicians take part in the planification of athletes' preparation^19^. Sports physicians could help trainers adjust training intensity when working with children or teenagers. R. È Motylânskaâ insisted on the necessity to combine a double gaze and a double expertise to improve sportsmen's performance^20^. Physicians were necessary to set training intensity, to protect children and teenagers' bodies and to enable them to reach higher performance levels. In a few words, R. È Motylânskaâ noted that intensive training could be harmful.

The 1962 issue also gave an example of how medical control and sports observation were used to determine what kinds of practices were possible. With regard to weightlifting, one article proposed to determine how this activity could be practiced by youngsters. Some physicians wanted to forbid it under age 17. A group of physicians from the Moscow Physical Culture Institute observed young weightlifters concluded that intensive training could be authorized after 15–16 years old. Nevertheless, they considered that it was possible only if sportsmen were practicing other physical activities (skiing, gymnastics) and closely supervised by trainers and physicians^21^.

The different works gathered in these two issues testified the evolution of sports research dealing with children and teenagers sports. Physiological and medical works gained in importance, as did tactical and training methodological processes. The age limit on intensive practice was discussed. Trials and observations were realized in child or youngsters training groups. In practice, this process permitted to legitimize child involvement in early specialization. In parallel, young sportsmen had to be protected. Interventions were necessary to protect them from traumas and disharmonious development. In doing so, Soviet physicians and specialists of training methods justified their specific intervention to fit with what Baptiste Viaud and Bruno Papin called the “double paradox of the body”: the necessity to increase its profitability and to protect it so as not to interrupt its productivity (Viaud and Papin, 2012).

The development of specific research on youth sports contributed to the institutionalization of a subfield in Soviet sports sciences and to the legitimization of adolescent involvement in elite sports. Conferences about the problems of youth sport were regularly held since 1964. A chair on theory and methodology on sport for children was created at the Moscow Institute in 1966 (Timakova, 2018). Research was launched not only in the central institutes. In the Tbilissi Institute of Physical Culture, scholars studied football for children and youngsters and developed joint research programs with scholars from the department of applied mathematics and cybernetics. In the Institute of Physical Culture of the Armenian SSR, scholars developed investigations about youths' visual and vestibular systems and pedagogical experiences^22^. In these studies, the age of the athletes observed by scholars was ever younger. In the introduction of the third scientific conference on the problems of child and youth sport held in 1973, Vladimir Pavlovič Filin, professor at the Moscow Sports Institute, summed up the development of the scientific field. Between 1954 and 1961, 260 PhDs were defended in sports sciences. Seventeen percent of them were on child sports and physical activities. Between 1962 and 1969, 495 PhDs were defended, in which 20% were on child sports^23^. Among the new topics that emerged in the 1960's, one of them was the direct result of a political demand: the early sorting of promising and talented sportsmen.

Tat'âna Timakova, in a book she published about selection in sports in 2018, recalled the major stages of research on early selection in Soviet sport. She pointed out the role of the applied research organized by V. P. Filin, S. S. Grošenkov and R. È Motylânskaâ, both in the GCOLIFK (State Central Order of Lenin Institute of Physical Education) and in the VNIIFK (All-Union scientific institute of physical culture). As noted, V. P. Filin and R. È. Motylânskaâ developed studies at the end of the 1950's to analyze the physiological effects of intensive training at various stages of life. They had also collected anthropometric data on youngsters involved in performance sports. V. P. Filin recalled that in 1963 the “Goskomsport” [the Central Union of Sports Associations and Organizations] asked the VNIIFK to develop research on selection in sports. This demand shows how it is necessary in the study of Soviet sports policies to not separate sports from other policies and other spheres of state intervention. This trend was visible in domains in which the Soviet motherland wanted to show its primacy (arts, music, mathematics) and needed high-skilled representants.

Since the mid-1950's, a network of specialist schools, “paths to excellence,” existed. To gain entry, a child had to prove his or her high level of ability (Dunstan, 1978). This was accelerated after the Tokyo Olympic Games, where the Soviet sportsmen faced growing competition^24^. By asking for this applied research, the Central Sports Administration wanted to rationalize and to optimize the selection process in sports and to give impetus to research dealing with this question. Consequently, in the VNIIFK, in the theory and methodology of child sports department, a special laboratory on sports selection was created, led by S. S. Grošenkov. The selection process was based first on physical and anthropometrical criteria, collected from contemporary champions and medalists. The selection process at a given child's age began to include psychological data and to analyze it by using statistics and computing (Timakova, 2018). By crossing physical, biomechanical and psychological information, scholars established the main characteristics needed in each discipline and fixed the selection on specific criteria.

The norms they established were then implemented and diffused in sports schools for the youth (DÛSŠ). A methodological letter was prepared and approved by the presidium of the All-Union Council of Sports Associations and Organizations in October 1965. The existence of these instructions shows two elements. First, the DÛSŠs, which tended at first to promote the practice of sports for youngsters, were included in the process of the making of the sports elite. Many reports in the Komsomol archives or the Committee archives complained about the difficulties faced by the DÛSŠs, their paucity and their inefficiencies^25^. This methodological letter aimed at reorganizing sports schools and at professionalizing their activities by setting up official guidelines based on a scientific approach. Secondly, the norms promoted the DÛSŠs as a key moment to select which child or teenager could be considered as promising and gave them a formative role. The norms also tried to regulate when a sports career could begin by determining the age limit to enter a DÛSŠ. The age to be admitted to a sports school varied from discipline to discipline. In swimming, the age limit was set at 7; in boxing, 14; in tennis, 9; in weightlifting, 15 (Table 1).

**Table 1:** Minimum age to be admitted in a sports school (GARF, f. 9570, inv. 1, d. 281, l. 76–77).

Alpine skiing	9
Artistic ice-skating	8
Chess	10
Football	11
Gymnastics	Girls,11; boys, 12
Rowing	13
Shooting	14
Table tennis	9
Track and field	12
Volley-ball	11
Water-polo	13

The entry selection process was divided into two steps: first, general physical activities permitted local coaches to spot children with physical abilities according to the grid prepared by physicians and scholars; secondly, children had to realize specific exercises, which permitted to sort them out between the different disciplines. The second step, according to the instructions, lasted between 3 and 6 months. Trainers looked for physical abilities and moral qualities, as well as working capacities and perseverance. During these months of observation, children were also under medical control and anthropometrical data was gathered by the medical staff: body proportions, limb length. This information was decisive to evaluate the potential growth of the body and its physical capacities. For some sports, such as diving, gymnastics and slalom, the medical staff evaluated the vestibular system of the children^26^. Tables with average anthropometrical data accompanied the letter to specify child physical characteristics in swimming, track and field, gymnastics, basketball and to make the process of selection easier (Table 2).

**Table 2:** Examples of anthropometrical characteristics in three disciplines for a 11-year-old girl (GARF, f. 9570, inv. 1, d. 281, l. 100–109).

	**Gymnastics**	**Swimming**	**Basket-ball**
Height (average/standard deviation)	138.962 (6,642)	143.620 (8,520)	149.258 (8,172)
Weight	31.524 (4,828)	35.968 (6,744)	40.774 (7,962)
Breast girth	65.696 (4,524)	68.642 (6,414)	72.532 (5,120)
Shoulder diameter	34.664 (10,591)	31.420 (3,554)	33.760 (3,830)
Spirometry	1,844.5 (636)	2,037.3 (491.2)	2,399.5 (615.5)
Back strength	52.844 (17,160)	46.450 (21,490)	43.240 (15,230)
Strength of the right hand	18.724 (6,968)	18.320 (6)	18.916 (4,492)

*NB, Units of measurement are unspecified in the original documents*.

Documents in the archives consulted did not provide further information on the way these tests were implemented or if this implementation was widespread. Criticism of the DÛSŠs remained vigorous. In *Sovetskij Sport*, as was frequently the case in the Soviet press, an article denounced the poor performances of the sports schools for the youth^27^. A report written in 1970 attested that the preparation in the DÛSŠs was not effective. If some had a good reputation and were renowned for the quality of the preparation (Voronej in volleyball, Alma-Ata and Tbilissi in wrestling), the problem of trainers' qualification remained; training was not well-planned; schools had difficulties in attracting young sportsmen, particularly in swimming and track and field; their action was not strongly coordinated, even if common patterns had been designed^27^.

At the beginning of the 1970's, the laboratory on sports selection disappeared during the reorganization of the VNIIFK. After the 1972 Olympics, criteria of selection were determined by each KCP (*Kompleksnyj celevyj Program*), a targeted program organized in each national selection, where scholars were associated with trainers. In the perspective of the Moscow Olympics, coaches continued to spot high-skilled youngsters but progressively gathered them in Olympic reserve centers (like *Krugloe Ozero* in gymnastics) where they were trained, cared for and observed (Timakova, 2018; Dufraisse, 2019). In 1978, 85 centers grouped 3,283 sportsmen and sportswomen^28^. In parallel, the network of sports boarding schools where children were admitted at the age of 11 or 12 expanded. Boarding school recruited children who did well in local games or were scouted, or selected children according to physical abilities and anthropometrical criteria (Dunstan, 1978). John Dunstan noted that some groups (teachers, ideologists, parents) expressed worries in *Sovetskij Sport* and underlined frequent dysfunctions: high dropping-out rates, badly designed curriculum, poor infrastructure, objections to sport as a career, neglect of mass sports… The depreciative visions of child elite sport, of the Soviet choice to develop it and its negative consequences on children's minds and education, also emerged in Soviet sports films.

### Discordant Appreciations and Visual Representations of Child Involvement in Elite Sports

Soviet film directors produced films about sports elites where they promoted the values and the ethos of the Soviet citizen since the 1930's. The narrative these films developed was not unique and was far from representative of a single model of the new Soviet men or women. The number of films about sports grew during the second half of the twentieth century in the USSR. Narratives of fictional and non-fictional films about champions were adapted to the existing ideological attempts and to the evolution of the Soviet society: the cult of Soviet champions in the context of the Cold War, the development of mass culture for the “Soviet baby boomers” and the multiplication of media where these films could be screened (cinemas, TV) (Dufraisse, 2019). It was also a way to fit public taste and contemporary issues^29^. In the 1970's, children and teenagers became heroes of fictional and documentary films on elite sports. This section examines eight films, four non-fictional and four fictional ones, released between 1968 and 1988, and illuminates how films revealed discordant representations of child elite sports in the Soviet Union. By juxtaposing the narratives of the films, this section notes criticisms that emerged in the Soviet Union at a moment when more children than ever were involved in elite sports.

In *Moj pervyj stadion* (Rybakova, 1970), *Dvoe na l'du*, (Grigor'ev, 1974) and *Sport strany sovetov* (Rybakova, 1979), the mass of young sportsmen *(massovost')* was the fundamental base to the top level (*master'stvo)*. The three films developed along the same patterns, portrayed pretty much the same images and gave coherence to the Soviet system of elite sports. They contributed to shaping common representations of how performance was obtained and to justify massive investments in elite sports. Children practiced in sports circles, in pioneer groups (a mass youth organization of the Soviet Union for children aged 9–15) and sports schools under the firm, precise but cheerful direction of their numerous coaches. “The goal was not to produce champions but to preserve the health of the next generations,” noted the narrator of *Sport strany sovetov* (Dufraisse, 2016). From this mass of young athletes, coaches selected the ones who had the best physical and moral abilities to train them more intensively and produce the actual champions. Each of the three films ended with the performances of the best Soviet champions, like Irina Rodnina, Sofia Muratova, Aleksandr Gorškov or Nikolaj Andriânov. These three documentary films developed the same “democratic fiction” (Fleuriel, 2004). They represented the same positive narrative of child involvement in sports: mass sports led to the elite. These films provide a qualified, evolutive vision of youth elite sport.

At the same time, some films presented a bitter image of children and teenagers' involvement in elite swimming and gymnastics. They pointed out the troubles that youngsters could suffer from when looking for records and performances. The film *The New Girl* [*Noven'kaâ*, (Liûbimov, 1968)] depicts how a young Muscovite girl, modern and carefree, Valentina Cernova, enters elite sports. As Valentina is talented, her coach, Anna Ivanovna, a former gifted gymnast, invites a senior coach to watch her performance. He recruits her in the training group in which the champion Ol'ga Kameneva also trains. Valentina spends her time in harsh training. As a result, she enters the national team. This film is centered on the trajectories of three women who were involved in elite sports careers. Coach Ivanovna is a friend of Ol'ga Kameneva's, they were raised in the same gymnastics school, but Ivanovna chose marital life and having kids over the possibility of a sports career. She now teaches gymnastics in a gloomy sports school. Ol'ga Kameneva is used to international podiums, but everyday training is now difficult for her and she has difficulties coping with its intensity, the concurrence and the more and more difficult acrobatics. Her sports career ends up in lassitude and doubt. Valentina is obliged to adjust the pattern of her life to gymnastics, to temper her behavior and to make sacrifices to be allowed the possibility of entering the national team. No heroism is here visible; only questions, doubts, bitterness and choices raised by a trajectory in elite sports emerge (Dufraisse, 2019).

Even if the film didn't get a large audience, Elem Klimov's *Sport, sport, sport*, released in 1970^30^, points out the panoptic control and the routinized life of sports boarding schools. Elem Klimov filmed a swimming school training. The film points out conflicting views about sports: children swim enthusiastically but are also tired and bored by the daily intensive training program. During their interviews, they reveal that they swim incredible distances every day: 3 km for a 4-year-old child, 15 km for a youngster (Makoveeva, 2002; Dufraisse, 2019).

The multi-prized film by Viktor Sadovski *A Moment Decides Everything* (*Vse reshaet mgnovenie*, 1978) also deals with the difficulties of elite sport. A gifted 15-year-old swimmer from a provincial city on the shore of the Black Sea is spotted by a national-team scout who invites her to train in an elite sports center. Used to swim in the sea with a dolphin as a hobby and to satisfy her own delight, the intense training regimen, the everyday concurrence in the national training center and the objective of the defense of the motherland make her stressed. At the European Championships, just before the relay team competition, she gives her place to a more-experienced champion because she has difficulties coping with the pressure. Even if she is able to overtake records, she is sent home to the Black Sea to relax. Coaches want to take care of their child prodigy. As Denise Youngblood and Tony Shaw demonstrate, this film illustrates that “maturity proves more important than raw talent” (Shaw and Youngblood, 2017).

This trend was also exploited in documentary films like in Rybakova's *Olympic hopes* released in 1978^31^. This film presents to the Soviet audience the expected champions of the Moscow Olympics: Maria Filatova, Elena Mukhina, Vladimir Markelov. It shows the intense, repetitive and rigorous training Soviet gymnasts are involved in. Some tiny girls are crying, others are tired and bored. The journalist asked Vladimir Markelov: “What is difficult for you? Physical tiredness or the moral responsibility?” The gymnast answered that he was used to physical fatigue and that the moral tiredness was the most difficult to cope with.

The film *Kukolka* was definitely the most critical. Released in 1988, it shows a situation far from the idealized picture that was described in the films evoked earlier. Isaac Fridberg's film presents a bitter vision, typical during the perestroika. A young woman trained to become a champion is severely wounded. She is obliged to come back to normal life and to the college after years traveling abroad, competing at a world level and reaching international podiums. The film highlighted the effect of high-level sports on individuality: poor education, insolence, attraction to material goods and to western bad influences… It illustrated the difficulties of young sportsmen, excluded from the sports career, in reintegrating normal life and in coping with it. But the discourse this film presented was not new. It rehabilitated the debate about the “star syndrome” that exploded in the Soviet public sphere around the “Streltsov scandal” at the end of the 1950's with the example of a young and female teenager, with the same arguments and remarks (Dufraisse, 2019). If some films, in the 1970's, demonstrated that children could be raised to the top and that sports could lead them to become ideal Soviet citizens, *Kukolka* shows the opposite: sports could be socially and bodily harmful for the youth and sportsmen and sportswomen could be negative examples. Feeling as an alien in an everyday life she was not used to, the “doll” ended up committing suicide (Shaw and Youngblood, 2017; Polivanova and Shakarova, 2018).

These films testify that in the USSR in the 1970's and the 1980's, the vision of elite sports was not monolithic. It also shows that debates about childhood evolved. Elizabeth White noted that the conception of what a child was changed between the 1950's and the 1980's. “Children were becoming regarded not just as self-sufficient future citizens, but also as vulnerable, unique individuals in need of special psychological protection” (White, 2020).

Obviously, these films did not capture the reality of Soviet youth elite sports in the 1970's but they contributed to giving it a shape, to perform principles and they attested that discordant visions of sports coexisted within the media. Some films we used as examples highlighted the difficulties of everyday training on bodies and minds and focused on the increasing pressure imposed by adults on young children. The Soviet Union cultural productions were not impervious to the debate that occurred in the international world of sports on child abuses in elite sports.

### The Rejuvenation of World Elite Sports and the Internationalization of the “Age Question”

During the 1970's, in the international world of sports (federations, media), the controversy about child and elite sports came to the forefront. In 1975, Ursula Weiss published an article about “Age and Peak Performance” in the *Olympic review*, which helped put the age issue on the agenda of international sports bodies. As Weiss wrote, “Nowadays, world swimming records are being broken by mere schoolgirls. In other sports too, peak performances are achieved at an increasingly early age, with the result that the age at which young hopefuls start serious training is becoming younger and younger^32^.” Two sports were particularly at the center of the attention: swimming and gymnastics. This section will retrace how child elite sports became an issue discussed in international federations and media.

In 1971, the International Gymnastics Federations (FIG) began to examine the question of age. Georgia Cervin indicates that the “assumption that Olga Korbut and Nadia Comaneci redefined women's gymnastics as a juvenile sport,” is overstated and that younger and acrobatic performers appeared in gymnastics at the beginning of the 1970's (Cervin, 2015). The FIG published articles in its bulletin to warn about the dangers of intensive youth training and blamed the production of “competitive animals.” As a consequence, the federation implemented age requirements to prevent girls younger than 14 from participating in elite competitions, a ruling that set the tone at the 1972 Munich Olympics. But the breaching of the rules continued as national federations were responsible for ensuring gymnasts were the required age (Cervin, 2015) without outside supervision. The Canadian Karen Kelsall was 13 when she competed at the 1976 Olympics. In July 1980 at the 58th FIG Congress, the minimum age was raised from 14 to 15. These requirements were applied in 1981. The Soviet prodigy Olga Bicherova was the revelation of the World gymnastics championships held in Moscow in 1981^33^. Years later, Bicherova admitted that she was younger and had competed with false documents (Riordan, 1993).

National federations had different strategies to enforce the new requirements. But the subversion of age rules began to create international controversies in the context of the renewed tensions between the USA and the USSR. It was invoked in the American press to reveal the socialist countries' duplicity and the double speech of the international federation of gymnastics. Neil Amdur focused his article about the 1981 Moscow gymnastics championships on the problem of underage gymnasts:

“The phenomenal success of young female gymnasts, some of whom may have been illegally entered in last month's world championships, has created controversy and division within the fast-growing sport. […] Two United States bronze medalists in Moscow, Julianne McNamara and Tracee Talavera, charged earlier this week that Olga Bicherova of the Soviet Union, who won the all-around title, was also under 15 […] Two years ago, Miss Talavera, then 13, was denied entry to the world championships in Fort Worth because of her age [She was 12.5]^34^.”

The mediatization of sports life and of sports celebrities contributed to highlighting tragic events and to giving them a worldwide resonance and signification. One heartbreaking accident in gymnastics at the beginning of the 1980's fueled the discussion about age requirements and child abuse in sports. Elena Mukhina beat the well-known Nadia Comaneci and won the all-around title at the 1978 World Championships in Strasbourg. Her gymnastics combined the elegant Soviet ballet style and new acrobatics that corresponded to the trend of “acrobatisation” of women's gymnastics. She was the first to realize a tucked double back salto dismount on beam or a full twisting double back somersault on floor. As she suffered from a broken leg, she was not able to take part in the 1979 World Championship in Texas. After the surgery, she returned to training to prepare for the Moscow Olympics. Her coach, Mihajl Klimenko wanted her to realize a dangerous new element that ended with a forward roll only men performed: the Thomas salto. Years later, she explained that she hurt herself several times during training. On July 3, 1980, just before the opening of the Olympics, the favorite Elena Mukhina suffered a tragic accident during training. She crash-landed and became instantly quadriplegic. Ten days later, on July 13, American journalist Barry Lorge wrote about her misfortune in the *Washington Post*. He evoked the fact that she crushed several vertebrae in her neck while practicing difficult acrobatic routines. The journalist noted that “her gymnastics career surely ended” and that this accident “must be one of the saddest stories” of the Moscow Games^35^. In *Le Monde*, on July 23, Alain Giraudo was far more elusive and critical toward the Soviets. He used the example of Elena Mukhina to describe the sensation of discomfort that followed the opening of the Moscow Games. “What happened to Elena Mukhina? […] We know she was terribly wounded during training at the beginning of July. So much for the facts. We don't know either on which apparatus she felt or the gravity of her injury (vertebrae could be affected) […] A rumor had been propagating in Moscow: Mukhina is said to be dead and, because of the Olympics, Soviet leaders didn't want to admit it.” Mukhina's tragic story became the symbol of abusive training programs. The race for the medals, wished by politicians and implemented by administrators and trainers, might injure Soviet children and teenagers' bodies and pushed them to their physical limits. Moreover, the Soviet Union sports authorities took some time revealing precisely how Mukhina's accident took place.

These polemics about children and teenagers' involvement in elite sports took place in a national and international configuration where the question of children's rights was evermore at the center of attention in professional communities as in broader media. In France, Jean Paulhac started the controversy about youngsters and endurance race, claiming for the stop of the “slaughter of innocents^36^” in *Le Monde* in 1975. In 1977, in the professional review *EPS*, the physical education teacher Jacques Personne pursued the controversy by arguing that the process of early specialization did not let children follow their own purpose and choices^37^. Baptiste Viaud has also underlined that this question divided the milieu of French sports physicians during the 1970's and the 1980's, between the supporters of the normalization of child elite sports and the opponents to intensive child practice (Viaud, 2009). In the United States, books began to openly criticize the consequences of child involvement in elite sports. In 1974, the reporter Marty Ralbovsky, in the book *Lords of the Locker Room*, condemned children's sports intensive training, the violation of young athletes' rights and the way adults harassed youngsters in their will to win (Ralbovsky, 1974). In 1978, the psychologist Rainer Marten published *Joy and Sadness in Children's Sports*. He proposed endorsement of a “Bill of rights of Young athletes” to protect the social and psychological health of children involved in sports (David, 2005).

Intergovernmental organizations worked specifically on sport for children, like the European Council (EC). The EC, which gathered Western European countries at the beginning of the 1980's, had a key role in the development of norms in Europe and was a precursor in the antidoping cooperation, in the promotion of sports for the disabled or in the struggle against violence in stadiums. The EC also cared about the involvement of youths in elite sports. In response to the recommendation of the 3^rd^ Conference of European Ministers Responsible for Sport held in April 1981, the Committee for the Development of Sport organized a September 1982 seminar on sport for children (6–12) that tackled the question: what effects do early specialization, intensive training and competition have on the psychological, physiological and social development of children^38^? “The seminar produced a number of practical conclusions which (i) aimed to foster the child's general and social development, and (ii) showed the physical and psychological dangers of subjecting children to the adult system of competition. It is now up to governments and sports federations to put these conclusions into effect^39^.”

During the second half of the twentieth century, the Cold War context and the intensification of the competition between the Soviet Union, countries from the Eastern and the Western bloc on pitches and stadiums contributed to modifying national sports systems and sportsmen's activity. It gave impetus to the extension and the rejuvenation of the sports career and Soviet organs faced this new trend. The Soviet sports administration developed a varied apparatus to include children and youths in elite sports. The will to take into account new considerations about the vulnerability of children, to improve the efficiency of the sports system and also to improve the abilities of high-skilled Soviets led to the development of scientific investigations on methods for training children, on the physiological consequences of intensive practice and on the physical qualities needed, on life-cycle in sports, on sorting processes. In doing so, scholars also contributed to specifying sports career according to the disciplines, to optimizing training, to increasing its profitability and to protecting young athletes, so as not to interrupt their productivity (Viaud and Papin, 2012).

Counterintuitively, from a Soviet point of view, the apparatus that resulted from this biopolitical expertise was designed to take care of the young sportsmen involved into elite sports. In more legitimized spheres, such as music or dance, analogous Soviet structures were admired, even in Western countries. But, even in the 1970's Soviet Union, dissonant discourses in the press or in films on child involvement pointed out the abuses of intensive children sports practices and of the investments that privileged elite sport and neglected mass sport. The excesses of Soviet early child involvement became the center of the international attention when the question of children's rights in sports arose at the end of the 1970's and at the beginning of the 1980's. Sports boarding schools, Soviet Olympic reserve centers and intensive practice imposed at a young age embodied the abuses of the “totalitarian” Soviet Union. It easily symbolized how the State molded its citizens to reach its goal, without paying attention to its citizens as subjects and individualities. Yet, it's necessary, while speaking about the controversies that arose during the Cold War about Eastern sports, to overcome the “cliché” of the “triumphalist Cold War narrative in the West,” blaming the multiple “abuses” of the socialist countries (Mellis, 2019), and to recontextualize them in the national and international context of their breaking.

## Author Contributions

The author confirms being the sole contributor of this work and has approved it for publication.

## Conflict of Interest

The author declares that the research was conducted in the absence of any commercial or financial relationships that could be construed as a potential conflict of interest.

## Footnotes

^1^AMDUR Neil, ≪ Rift over underage gymnasts”, *New-York Times*, December 7, 1981, p. 4; “Olga Bicherova, who a month ago would have been too young to compete…”, *United Press*, November 28, 1981.

^2^MILŠTEJN Oleg Aleksandrovič, KULENKOVIC Konstantin Antonovič, *Sovetskij Olimpiec*, op. cit., p. 26.

^3^*Rossijskij gosudarstvennyj arhiv social*′*no-političeskoj istorii* (RGASPI), f (m)1, o. 38, d. 206, p. 17-87.

^4^RGASPI, f (m)1, o. 38, d. 206, p. 17.

^5^*Gosudarstvennyj arhiv Rossijskoj Federacii*, (GARF), f. R7576, inv. 1, d. 578, l. 117.

^6^GARF, f. R7576, inv. 1, d. 760, l. 71.

^7^RGASPI, f. m1, inv. 47, d. 392, l. 42-43.

^8^BATYROV X. A., Razvitie ûnošeskogo sporta v SSSR, 1946-1970 [dissertation thesis], Moscou, State Central Physical Culture Institute, P. 8.

^9^GARF, f. R9570, inv. 1, d. 94, l. 55.

^10^GARF, f. R9570, inv. 1, d. 269, l. 27-30.

^11^GARF, f. R7576, inv. 31, d. 747, l. 152.

^12^GARF, f. R7576, inv. 29, d. 175, l. 117.

^13^GARF, f. R7576, inv. 29, d. 175, l. 3.

^14^GARF, f. R7576, inv. 29, d. 176, l. 14-15.

^15^GARF, f. R7576, inv. 29, d. 182, l. 117-120.

^16^*Rossijskij gosudarstvennyj arhiv novejšej istorii*, (RGANI), f. 5, inv. 76, d. 209, l. 8; GARF, f. R9570, inv. 1, d. 14, l. 64.

^17^*Rossijskij gosudarstvennyj arhiv social*′*no-političeskoj istorii*, fM. 1, inv. 47, d. 67, l. 17.

^18^*Problemy ûnošeskogo sporta*, 1, 1958, p. 3.

^19^*Problemy ûnošeskogo sporta*, 2, 1961, p. 231.

^20^*Problemy ûnošeskogo sporta*, 2, 1961, p. 156.

^21^*Problemy ûnošeskogo sporta*, 3, 1962 p. 222-223.

^22^*Sbornik dokladov na 2oj Vsesoûznoj Konferencii po problemam ûnošeskogo sporta*, Metodičeskij kabinet GCOLIFK, 1974, p. 9-10.

^23^*Tezisi dokladov 3oj vsesoûnoj naûčnoj konferencii po problemam ûnošeskogo sporta, Moscou*, 1973, p. 3.

^24^GARF, f. 9570, o. 1, d. 281, l. 68.

^25^RGASPI, f. m1, inv. 47, d. 392, l. 42-43; GARF, f. R9570, inv. 1, d. 270, l. 52.

^26^GARF, f. 9570, o. 1, d. 281, l. 68-75.

^27^GARF, f. R7576, inv. 31, d. 44, l. 7.

^28^GARF, f. R7576, inv. 31, d. 4797, l. 1.

^29^To understand the functioning of post-war Soviet cinema industry: Tcherneva, I. (2014). Le cinema de non-fiction en URSS. Création, production et diffusion (1948-1968). [dissertation thesis], Paris, EHESS.

^30^KLIMOV Elem, *Sport, Sport, Sport*, 1970, 85 min.

^31^RYBAKOVA Alexandra, *Olimpiskie nadeŽdy*, 1978, 19'34.

^32^WEISS Ursula, ≪ Age and Peak Performance”, Olympic Review, 89-90, 1975, p. 106-114.

^33^ALBOUY Gérard, “Mainmise soviétique sur les championnats du monde”, *Le Monde*, December 1, 1981.

^34^AMDUR Neil, ≪ Rift over underage gymnasts”, *New-York Times*, December 7, 1981, p. 4.

^35^LORGE Barry, “Mukhina Badly hurt in Gym Fall”, *The Washington Post*, July 14, 1980.

^36^PAULHAC Jean, ≪ Le massacre des innocents ≫, *Le Monde*, May 10, 1975.

^37^PERSONNE Jacques, ≪ Il faut arrêter le massacre des innocents ≫, *revue EPS*, mars-avril 1977, p. 18-19.

^38^Council of Europe, Committee for the development of sport, sport for children, Tonsberg (Norway), 27 september-1 october 1982, Invitation and draft programme, Strasbourg: Council of Europe, 1982. CDDS(82)1_eng_COE155662.

^39^Council of Europe, Committee for the development of sport, Annual Report for 1981 and 1982, Strasbourg: Council of Europe, 1982. COE163572.
